# First person – Sophie Badger

**DOI:** 10.1242/dmm.052304

**Published:** 2025-02-13

## Abstract

First Person is a series of interviews with the first authors of a selection of papers published in Disease Models & Mechanisms, helping researchers promote themselves alongside their papers. Sophie Badger is first author on ‘ A bacterial artificial chromosome mouse model of amyotrophic lateral sclerosis manifests ‘space cadet syndrome’ on two FVB backgrounds’, published in DMM. Sophie conducted the research described in this article while a PhD student in James Alix's lab at the University of Sheffield, Sheffield, UK. She is now a postdoctoral research associate in the lab of Guillaume Hautbergue at the University of Sheffield, investigating a potential gene therapy for *C9orf72*-associated amyotrophic lateral sclerosis (ALS)/frontotemporal dementia (FTD).


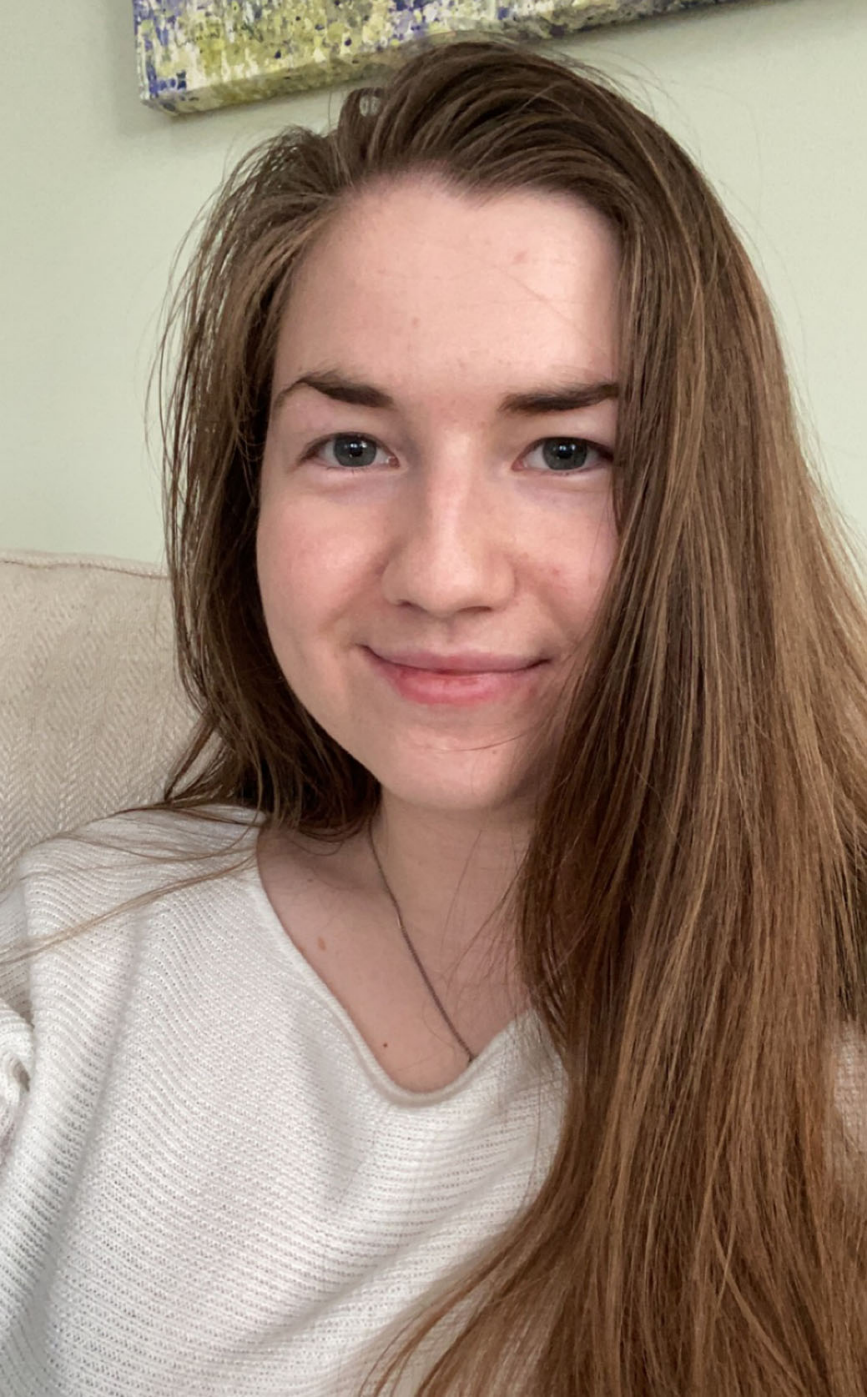


**Sophie Badger**

**Who or what inspired you to become a scientist?**

Ever since I was a young child, I have always had a deep interest in science, particularly biology. How the human body functions in its complexity and how diseases develop when these functions go wrong was a keen area of interest, and led me to check out as many books as possible from my local library that were even remotely related to biology throughout my childhood. Both my family and my biology teacher in secondary school fostered this deep-seated curiosity and encouraged me to pursue my interest all the way to university. While studying for my undergraduate degree, I chose a project on the neurodegenerative disease Huntington's disease for my final-year dissertation. This sparked my interest in the study of neurodegenerative diseases, while a lab placement over the summer gave me a taste for research in an academic laboratory environment. All in all, this cemented my desire to enter the world of neurodegenerative disease research and led to where I am today.

**What is the main question or challenge in disease biology you are addressing in this paper? How did you go about investigating your question or challenge?**

*C9orf72*-related ALS/FTD, also known as motor neuron disease (MND), has proven difficult to model in mice. In 2016, a new model was published – a bacterial artificial chromosome (BAC) transgenic mouse that manifested striking behavioural, motor and pathological abnormalities (Liu et al., 2016). However, this was followed by multiple laboratories independently both refuting and confirming these phenotypes. A proposed explanation for these conflicting results centred on the use of different FVB background lines of mice. Our study aimed to study this *C9orf72* BAC mouse model on both backgrounds to determine whether mouse background strain affected the striking neurodegenerative phenotype previously observed.

To investigate this, we performed extensive 12-month-long behavioural studies on *C9orf72* BAC mice on both backgrounds, and while we found significantly elevated levels of dipeptide repeat proteins (a toxic protein aggregate produced by the transgene), there was no evidence of a transgene-associated behavioural phenotype. We also observed seizures and a gradual decline in functional performance in transgenic and non-transgenic mice, regardless of genetic background. This phenotype had previously been reported in the literature and had been dubbed ‘space cadet syndrome’, where mice develop neurodegenerative-like behavioural phenotypes and hippocampal neurodegeneration. The results raise questions about the reported phenotype and highlight the importance of using genetic backgrounds which do not confound interpretation of neurodegenerative phenotypes.

**How would you explain the main findings of your paper to non-scientific family and friends?**

Animal models are a crucial tool used to study human disease. Animals like mice and rats are similar to humans, both genetically and physiologically, and allow us to study various diseases. They help us to improve our understanding of how diseases develop and progress, and ultimately aid in our goal to develop treatments.

The mouse model used in this paper was generated to mimic MND by giving them a DNA mutation that is known to cause the disease in humans. Initially, it seemed to be a very promising model that developed many of the symptoms seen in humans with MND. However, other labs that began working with this model did not find the same results and instead showed that the model did not display any symptoms of MND. Because of these contradictory results, our work aimed to determine why these labs were finding opposing results and whether or not the model truly did or did not develop MND symptoms. To do this, we obtained two different versions of the model: one from a lab that had observed MND symptoms in the model, and the other from a lab that had not seen symptoms. We then performed what is called a characterisation of the model, where we tested the ability of the mice to do various tasks such as how long they could balance on a rotating beam, took measurements from their muscles to determine muscle health and whether any degeneration was occurring, and examined their tissues after death to look at neuron health in the brain and spinal cord. We compared the mice that had the mutation that causes disease with healthy, control mice that did not have the mutation.

From our results, we concluded that neither model developed any symptoms of MND and that there was no difference between the two versions of the model. Instead, the presence of seizures and a general decline in the ability of all mice to perform the behavioural tests led us to believe that the strain of mouse used to create the model may have been affecting the results.

This research highlights the importance of mouse background strain when developing disease models.

**What are the potential implications of these results for disease biology and the possible impact on patients?**

This research highlights the importance of mouse background strain when developing disease models. It also provides further evidence that this *C9orf72* BAC model is unsuitable for studies based on behavioural readouts but could still be useful to study certain aspects of the pathology, such as the toxic dipeptide repeat proteins produced by the mutation. Developing reliable and reproducible disease models are vitally important for the study of human disease as they allow us to develop and test potential therapeutics that may ultimately go forward into clinical trials for patients.

**Figure:**
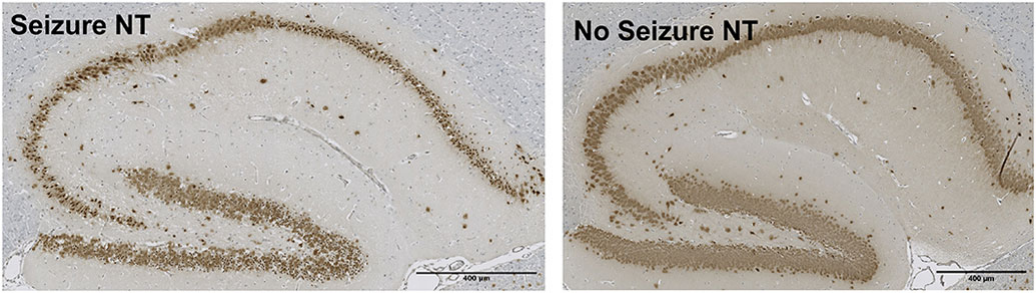
**Immunohistochemical staining of the dentate gyrus in the hippocampus of non-transgenic (NT) mice with and without seizures.** Marked neurodegeneration is observed in the NT mouse with seizures (left), similar to that seen in the transgenic mice of the initial publication of this *C9orf72* BAC model (Liu et al., 2016).

**Why did you choose DMM for your paper?**

DMM is a highly regarded, open source journal that publishes research on human diseases using various model systems. Our research was heavily focused on the characterisation of a disease model of MND, and so DMM was a natural home for the publication of our research.

Job insecurity and the resulting financial instability is an issue for early-career researchers like myself.

**Given your current role, what challenges do you face and what changes could improve the professional lives of other scientists in this role?**

Job insecurity and the resulting financial instability is an issue for early-career researchers like myself. Competition for grants is fierce, and more funding options and sources are required to allow for important research to continue. This can lead to many incredible and talented researchers jumping ship from academia to industry – or into different fields entirely – in order to secure more lucrative positions and opportunities. I love the work I do, and I would love to continue it for years to come. However, job insecurity is a real concern of mine, and if the current system doesn't change then a position in academia will no longer be viable, and I will be looking for opportunities in industry and beyond.

**What's next for you?**

I have begun my second postdoctoral position at the Sheffield Institute for Translational Neuroscience in Professor Guillaume Hautbergue's lab, where I am currently working on a potential therapeutic for *C9orf72*-associated ALS/FTD.

**Tell us something interesting about yourself that wouldn't be on your CV**

I am an aerialist! I've been attending aerial hoop lessons for over 2 years now and recently performed in a show.
